# Proteomic analysis of pleomorphic dermal sarcoma reveals a fibroblastic cell of origin and distinct immune evasion mechanisms

**DOI:** 10.1038/s41598-024-62927-x

**Published:** 2024-05-31

**Authors:** Sebastian Klein, Yuri Tolkach, Hans Christian Reinhardt, Reinhard Buettner, Alexander Quaas, Doris Helbig

**Affiliations:** 1 Department of Hematology and Stem Cell Transplantation, University Duisburg-Essen, University Hospital Essen, Hufelandstraße 55, 45147 Essen, Germany; 2 West German Cancer Center Network, Partner Site Essen, Essen, Germany; 3 Institute for Pathology and Neuropathology, University Hospital and Medical Faculty Cologne, Kerpenerstr 62, 50937 Cologne, Germany; 4 Center for Molecular Medicine Cologne, Cologne, Germany; 5 Department of Dermatology, Medical Faculty, University Hospital Cologne, Cologne, Germany

**Keywords:** Pleomorphic dermal sarcoma, PDS, Protein profiling, Fibroblastic differentiation, Immune escape mechanisms, Cutaneous sarcoma, Sarcoma, Skin cancer, Tumour biomarkers

## Abstract

Pleomorphic dermal sarcomas are infrequent neoplastic skin tumors, manifesting in regions of the skin exposed to ultraviolet radiation. Diagnosing the entity can be challenging and therapeutic options are limited. We analyzed 20 samples of normal healthy skin tissue (SNT), 27 malignant melanomas (MM), 20 cutaneous squamous cell carcinomas (cSCC), and 24 pleomorphic dermal sarcomas (PDS) using mass spectrometry. We explored a potential cell of origin in PDS and validated our findings using publicly available single-cell sequencing data. By correlating tumor purity (TP), inferred by both RNA- and DNA-sequencing, to protein abundance, we found that fibroblasts shared most of the proteins correlating to TP. This observation could also be made using publicly available SNT single cell sequencing data. Moreover, we studied relevant pathways of receptor/ligand (R/L) interactions. Analysis of R/L interactions revealed distinct pathways in cSCC, MM and PDS, with a prominent role of PDGFRB-PDGFD R/L interactions and upregulation of PI3K/AKT signaling pathway. By studying differentially expressed proteins between cSCC and PDS, markers such as *MAP1B* could differentiate between these two entities. To this end, we studied proteins associated with immunosuppression in PDS, uncovering that immunologically cold PDS cases shared a “negative regulation of interferon-gamma signaling” according to overrepresentation analysis.

## Introduction

Pleomorphic dermal sarcomas (PDS) are rare cutaneous malignant tumors; however, they are the most frequently observed among cutaneous sarcomas^1^. Within the head and neck region, PDS is the most commonly diagnosed sarcoma, accounting for approximately 40% of such cases^2,3^. Characteristically, these tumors are diagnosed in ultraviolet (UV)-exposed locations of elderly individuals with a risk of local or distant metastasis in approximately one third of these patients^4–6^.

For decades, there was a controversy whether PDS represent fibroblast- or myofibroblast-derived sarcomas or alternatively, dedifferentiated squamous cell carcinomas due to some genetic similarities – which may be also found in a plethora of other malignancies^7–9^.

By performing comparative analyses between PDS and other common UV-induced skin tumors, we demonstrated that PDS significantly differed from cutaneous squamous cell carcinomas (cSCC), cutaneous basal cell carcinomas (BCC) and malignant melanomas (MM) in terms of their different mutational frequencies^1^. Moreover, transcriptome sequencing with subsequent hierarchical clustering of gene expression profiles, as well as local mutation rate pattern analysis and immunohistochemical analyses allowed clear separation of PDS from cSCC. PDS clustered as a distinct entity with a defined mutational landscape, compatible with a mesenchymal fibroblastic differentiation of PDS, as opposed to an epithelial differentiation of cSCC and cBCC, and the neural crest origin of MM^1^. Furthermore, our investigations revealed that PDS harbor a high mutational burden with a genetic UV signature, and that the majority of these tumors are immunologically inflamed/immunogenic tumors with a high amount of tumor-infiltrating lymphocytes and expression of diverse checkpoint molecules, such as PD-L1, LAG-3, and TIGIT^1^. Based on these findings, we described the first PDS patients successfully treated with immune checkpoint blockade (anti-PD1 antibody Pembrolizumab)^1,10^.

The aim of the current study was to confirm the fibroblastic differentiation of PDS in comparison to other UV-induced tumors by performing proteomic analyses and highlighting potential mechanisms of immune evasion in PDS.

## Results

### Proteomic analysis confirms an association of pleomorphic dermal sarcoma with a fibroblastic cell of origin

**Figure 1:**
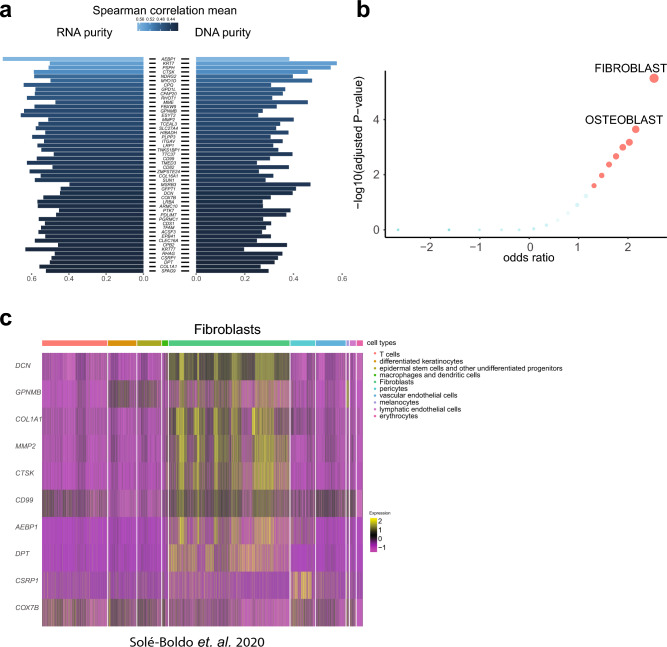
Correlation of combined tumor purity to protein abundance in PDS. (**a**) Bar chart of both RNA- (n = 21) and DNA- (n = 24) based purity calculation for PDS cases and the top 50, most correlating proteins to tumor purity, by following a Spearman correlation of each protein with tumor purity. The bar length corresponds to a Spearman correlation coefficient of an individual protein (visualized as human genome organization gene symbol; row), the color is coded for the mean correlation between both RNA and DNA correlation to tumor purity. The scale bar visualizes the mean Spearman correlation coefficient. (**b**) Scatter plot of an Enrichr^11^ analysis using the “ARCHS4 tissue” database of the top 50 proteins most correlating to tumor purity. The color code represents significant findings (FDR < 0.05, red) (n = 24). The adjusted p-value, the odds ratio and the log2 of the combined score (dot size) are quantitative and qualitative results of the Enrichr overrepresentation analysis, which briefly reflect the relevance of a given term in association to the proteins (here, we used corresponding HUGO symbols as input). (**c**) Heatmap of most highly expressed 10 genes, according to average gene expression within the single cell data^12^, which were also among the top 50 genes most correlating to RNA/DNA tumor purity of panel A (each column represents one cell, total samples n = 5).

Having previously provided evidence suggesting a fibroblastic cell of origin in PDS^1^, we wanted to confirm our findings, using proteomic analysis. We therefore analyzed 24 samples of the previously described cohort that had sufficient material for mass spectrometry. We then estimated tumor purity using both DNA-sequencing data, as described previously^1^, in addition to purity calculated based on RNA-sequencing data. We then ranked the top 50 correlating genes by averaging the correlation between both RNA- and DNA-based tumor purity and protein abundance (Fig. 1a).

In our study, we utilized estimates of tumor purity derived from both DNA and RNA-sequencing data. By correlating these estimates with protein abundance, our aim was to identify molecules predominantly expressed by the tumor cells themselves, potentially revealing markers indicative of the cell of origin. A notable outcome of this approach was the identification of PDGFRB using our proteomic dataset, a discovery we have also previously documented and further validated at both RNA and protein levels using immunohistochemical analysis^1^. In addition, by following a gene-set enrichment analysis using Enrichr^11^ and the ARCHS4 tissue database analysis, we could confirm that proteins most correlating to tumor purity were associated with fibroblasts (Fig. 1b).

Next, we explored single-cell sequencing data of a previous study analyzing healthy dermal tissue of five individuals^12^. Following the annotation of the published dataset of the individual cell clusters (Fig. S1B), we used the top 50 proteins correlating to tumor purity (Fig. 1a) and queried the 10 genes showing the highest average gene expression in the single cell data. In line with our observation using an unbiased overrepresentation analysis using Enrichr^11^ (Fig. 1b), we confirmed that most proteins identified by us in the tumor cell purity correlation analysis were associated to dermal fibroblasts in this dataset (Fig. 1c, top 10 genes visualized).

### Pleomorphic dermal sarcomas show activation of the PDGFRB-PDGFD axis

**Figure 2:**
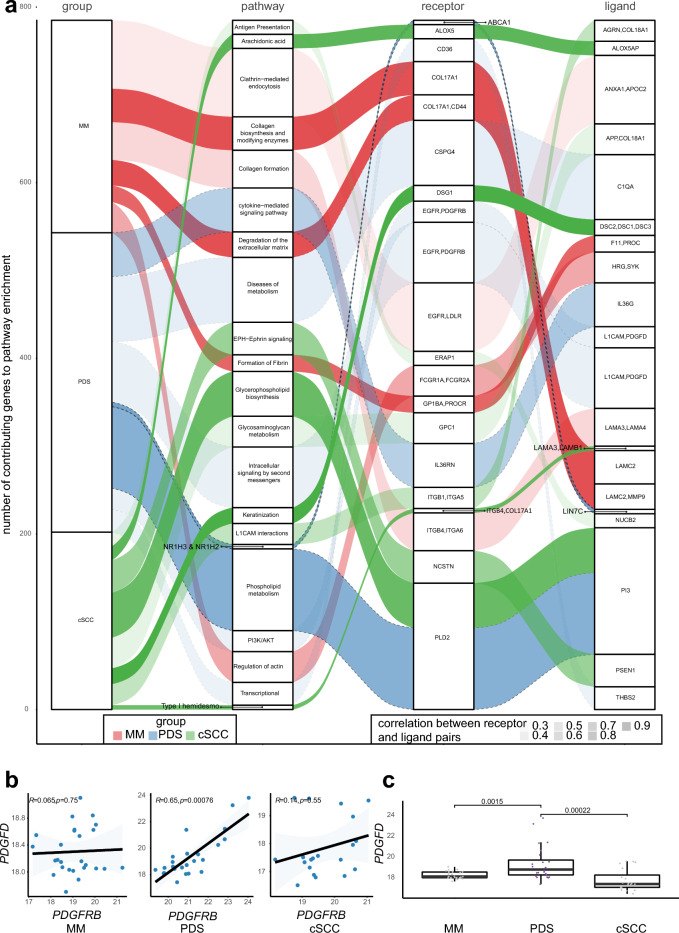
Upregulation of the PI3K/AKT signaling pathway in PDS by a PDGFRB-PDGFD axis. (**a**) Alluvial diagram of corresponding R/L interactions for malignant melanoma (MM), pleomorphic dermal sarcoma (PDS) and cutaneous squamous cell skin cancer (cSCC). The y-axis corresponds to the number of contributing genes to each enriched Reactome pathway^52^. For readability, we have abbreviated R/L interactions, a full list can be found in Table S3. (**b**) Scatter plots of Spearmen correlation for PDGFRB and PDGFD for each entity separately. (**c**) Boxplot for comparison of PDGFD abundance for MM (n = 27), PDS (n = 24) and cSCC (n = 20) using a compared using a Wilcoxon Rank Sum Test.

To study relevant associations of receptor-ligand interactions and downstream signaling pathways, we analyzed the proteomic data of PDS, malignant melanoma (MM) and cutaneous squamous cell carcinoma (cSCC) using a previously published method, which analysis relevant R/L and pathway interactions and triplets (receptor, ligand and Reactome/gene ontology pathways), and includes a statistical model correcting for random correlations^13^. After correcting for multiple testing (false-discovery-rate; FDR < 10^–3^), we uncovered distinct signaling networks for cSCC, PDS and MM (Fig. 2a). The strongest correlation of a signaling network, according to entity were “Keratinization” in cSCC (Spearman correlation = 0.9, FDR < 10^–10^), “NR1H3 & NR1H2 regulate gene expression linked to cholesterol transport and efflux” in PDS (Spearman correlation = 0.7, FDR = 3 × 10^–4^) and “degradation of extracellular matrix” in MM (Spearman correlation = 0.77, FDR = 5 × 10^–4^). There was no overlap between the enriched downstream signaling pathways of the given receptor-ligand interactions within the three entities of PDS, cSCC, and MM, given the threshold of an FDR below 10^–3^.

However, having previously shown that PDGFRB is highly expressed on PDS tumor cells, but neither on differentiated or undifferentiated (sarcomatoid) cSCC^1^, the enrichment of “PI3K/AKT signaling in cancer” in PDS (Spearman correlation = 0.29, FDR = 3 × 10^–4^), which included PDGFRB as a receptor and PDGFD a as ligand, was of interest. We therefore compared the correlation of PDGFRB and PDGFD between MM, PDS and cSCC. Here, only PDS showed a significant correlation between PDGFRB and PDGFD expression (Spearman correlation = 0.65, p = 7.6 × 10^–4^; Fig. 2b). In addition, PDS showed significantly higher PDGFD protein levels, as compared to MM and cSCC (Fig. 2c). We again explored single cell sequencing data of human dermal tissue^12^ and compared dedicated receptor/ligand pairs of PDS and cSCC (Fig. 2a; Table S3) – two tumor entities that shared some genetic mutation profiles^1^—to identify cell populations that would co-express both markers (Fig. S2). Informed by a significant correlation between the receptor/ligand pairs of PDGFRB and PDGFD in PDS, and a significant correlation between DSG1 and DSC1 in cSCC (Fig. 2a; Table S3), using our proteomic data, we explored whether these genes were also highly expressed in the given cellular compartments of the single cell data of the healthy dermal tissue. Interestingly, we could observe a co-expression of PDGFRB and PDGFD in fibroblasts, while DSG1 and DSC1 co-expression was only observed in keratinocytes (Fig. S2).

### Distinct clustering of pleomorphic dermal sarcoma in comparison to other common skin malignancies

**Figure 3:**
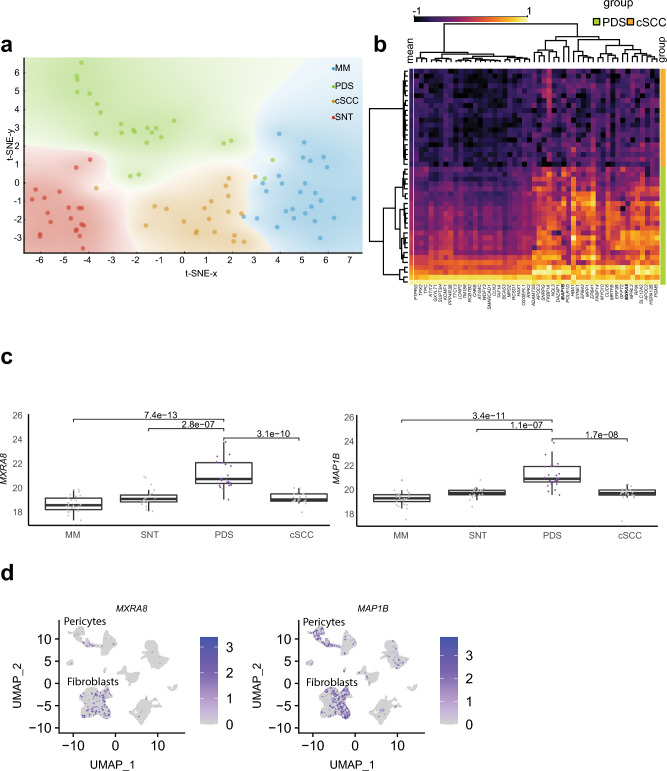
Distinct clustering of pleomorphic dermal sarcomas and potential protein markers to distinguish PDS from other skin malignancies*.* (**a**) t-SNE plot of malignant melanoma (MM), pleomorphic dermal sarcoma (PDS), cutaneous squamous cell skin cancer (cSCC) and skin normal tissue (SNT). The color represents the regional probabilities of being associated to one of the entities. (**b**) To identify proteins that differed specifically between cSCC and PDS, the data were normalized, scaling expression values between − 1 and 1. Then, a ranking process was employed based on two metrics: the chi-squared (χ^2^) statistic and the information gain ratio (IGR). The χ^2^ statistic evaluated the observed frequencies of protein count differences between the groups, while the IGR quantified the information a protein provides about the differentiation between the groups of cSCC and PDS. Using these criteria, the top 50 genes showing the most pronounced differences in abundance were visualized using a heatmap. Columns corresponded to proteins and rows to individual samples. Human genome organization gene symbols are used for annotating the proteins (cSCC, n = 20; PDS, n = 24). (**c**) Boxplot comparing MM (n = 27), PDS (n = 24) and cSCC (n = 20) using a compared using a Wilcoxon Rank Sum Test. (**d**) Feature plot (UMAP) of single cell sequencing data^12^ of skin normal tissue (SNT) for both *MXRA8* and *MAP1B*. Clusters of both fibroblasts and pericytes are highlighted (originated from n = 5 samples of human skin tissue).

By expanding our proteomic analysis to malignant melanoma (MM), squamous cell carcinoma (cSCC) and skin normal tissue (SNT), we wanted to compare the similarity of these entities to PDS. We therefore used the proteins most correlating to tumor purity (Fig. 1a) and visualized these data using a t-distributed stochastic neighbor embedding (t-SNE) comparing the distribution of the skin malignancies (Fig. 3a). Here, cSCC, MM and SNT clustered distinctly to PDS, forming a broad separate cluster, confirming our results using mutational patters using DNA sequencing^1^.

We then explored differentially expressed proteins between PDS and cSCC, given the hypothesis of a mesenchymal/fibroblastic cell of origin in PDS. By visualizing the top 50 proteins that were upregulated in PDS, we could observe that both PDS and cSCC clustered as distinct entities (Fig. 3b). By comparing a subset of these proteins, which were highly expressed in PDS, we confirmed that the upregulation of *MXRA8* and *MAP1B* were statistically significant in PDS (Fig. 3c; both p < 0.001). By again exploring the single cell sequencing data of dermal tissue, we could confirm that both *MXRA8* and *MAP1B* were mostly expressed by clusters annotated as dermal fibroblasts (Fig. 3d).

### Distinct repertoire of immune-regulating proteins in inflammatory cold cases of PDS

**Figure 4:**
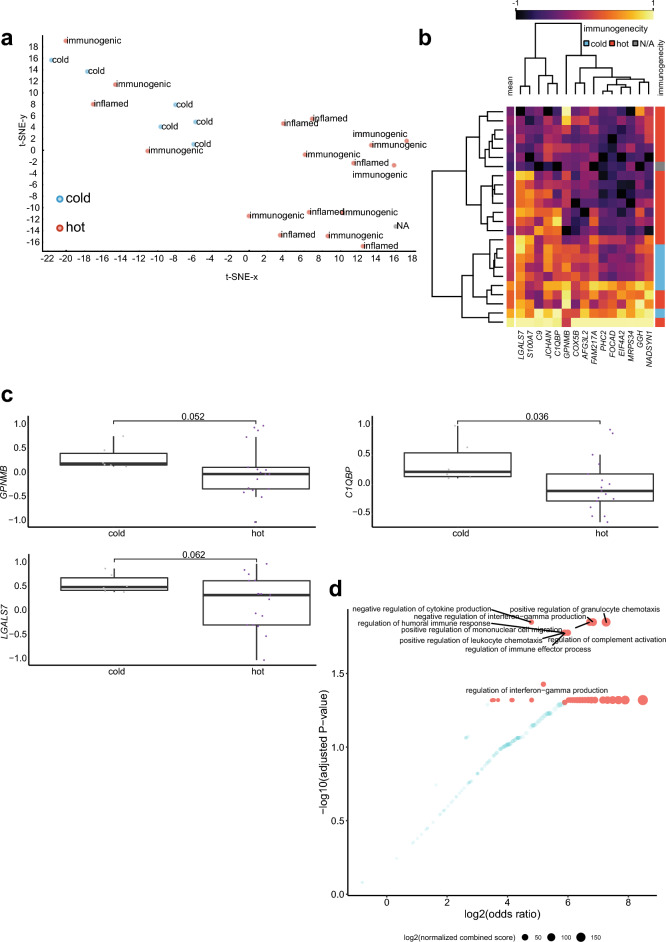
Comparing protein abundance associated with immunosuppression in PDS. (**a**) t-SNE plot of PDS cases comparing the immunological status using T-cell infiltration and PD-L1 expression. “Cold” tumors showing neither CD4/CD8 T-cell tumor infiltration nor PD-L1 expression; “hot” tumors can be divided into “immunogenic” tumors showing either PD-L1 expression or infiltration by CD4/CD8 T-cells or “inflamed” tumors showing both PD-L1 expression and infiltration by CD4/CD8 T-cells^1^ (**b**) Heatmap of markers separating inflammatory “hot” and “cold” PDS cases using Human Genome Organization gene symbols. (**c**) Box plot comparing dedicated markers between inflammatory “hot” (n = 17) and “cold” (n = 6) PDS cases (n = 24; one case not available/NA) compared using a Wilcoxon Rank Sum Test. (**d**) Scatter plot of an Enrichr^11^ analysis using the “GO Biological Process 2021” database of the top 50 proteins correlating to tumor purity. The color code represents significant findings (FDR < 0.05, red) (n = 24). The adjusted p-value, the odds ratio and the log2 of the combined score (dot size) are quantitative and qualitative results of the Enrichr overrepresentation analysis, which briefly reflect the relevance of a given term in association to the proteins (HUGO symbols were used as input).

We previously showed that PDS is a predominantly inflamed tumor entity, with several lines of evidence suggesting that immune checkpoint inhibition is a promising therapeutic option in PDS. However, according to our analysis, a subset of PDS cases appears to be immunologically “cold”, showing no PD-L1 expression and no infiltration of immune cells^1^. We therefore compared proteins that were associated to either “cold” or “hot” (which we defined as tumors not classified as “cold”) tumors. Indeed, we could separate inflammatory “cold” from “hot” tumors (Fig. 4a). Interestingly, there was a fraction of proteins that was increased in cases categorized as immunologically “cold” (Fig. 4b).

For a subset of these proteins, namely *GPNMB*, *C1QBP* and *LGALS7*, there was a statistical trend of being elevated in inflammatory “cold” PDS cases (Fig. 4c), suggesting a potential immunosuppressive role in this entity. We therefore used an unbiased approach of a gene-ontology analysis of proteins enriched in inflammatory cold tumors. Here, “negative regulation of interferon-gamma production [GO: 0032689]” and “negative regulation of cytokine production [GO:0001818]” were associated with proteins elevated in inflammatory “cold” tumors, by following an overrepresentation analysis using Enrichr (Fig. 4d).

## Discussion

Within our current proteomic analysis of pleomorphic dermal sarcoma (PDS), we have confirmed a distinct clustering of PDS in comparison to other UV-induced skin tumors, including malignant melanoma (MM) and cutaneous squamous cell carcinoma (cSCC). We have identified several proteins that were highly expressed within PDS tumors and showed a significant correlation with markers of tumor purity.

In particular, matrix remodeling-associated protein 8 (*MXRA8*) and microtubule-associated protein 1B (*MAP1B*) are both shown to be expressed by dermal fibroblasts or cancer‐associated fibroblasts in single cell sequencing analyses^14^. These molecules were significantly upregulated in PDS compared to MM and cSCC. High expression of MXRA8 has been associated with an unfavorable prognosis in glioma^15^ as well as in breast cancer^16^ whereas MAP1B in urothelial carcinoma of the upper tract and the bladder^17^. Besides, a higher MXRA8 expression correlated with immunoinhibitory molecules, such as TGF-b1, IL-10, PD-L1, and CTLA4 in glioma^15^.

By comparing inflammatory “cold” with “hot” tumors, the proteins *GPNMB*, *C1QBP* and *LGALS7* were significantly elevated in inflammatory “cold” PDS. GPNMB is an endogenous type 1 transmembrane glycoprotein that is constitutively expressed in most cell types and tissues^18^. It has been reported to be overexpressed in malignant tumors including glioma^19^, hepatocellular carcinoma^20^, rectal cancer^21^, malignant melanoma, head and neck cSCC and breast cancer^22–27^ as well as most sarcomas^28^. In most of these tumors, overexpression correlates with cancer aggressiveness, metastatic disease, and immunosuppression^22–25,29,30^. In melanoma cells, GPNMB attenuates the activation of T cells that are activated via syndecan-4, allowing the evasion of melanoma cells from immunological recognition and destruction^25^. Blocking GPNMB by antibodies restores the integrity of T cells, attenuates tumor growth, and increases interferon-gamma levels in the tumor microenvironment^31^. The interaction between Syndecan-4 and GPNMB further leads to a reduction of proinflammatory cytokine secretion and hinders T cells from entering the S phase of the cell cycle^32^. Following these lines of evidence, a mouse MC38 colon adenocarcinoma cell line that was made resistant to anti-PD-1, transcriptomic profiles revealed that *Gpnmb* was significantly upregulated in anti-PD-1 therapy-resistant compared to -sensitive tumors. Furthermore, deletion of *Gpnmb* in resistant cells successfully restored sensitivity to anti-PD-1 treatment in vivo^33^. It has also been shown that higher circulating GPNMB blood levels of patients with advanced non-small cell lung cancer and malignant melanoma were associated with resistance to immune checkpoint blockade^32,34^. Taking together our results and the studies described here, GPNMB could be a marker of poor prognosis in PDS. Future studies may need to investigate whether GPNMB could qualify as a biomarker for the treatment with PD-L1 inhibitors in PDS. Although these results are promising, they warrant further validation, especially given that the differences between inflammatory status of PDS tumors remained at a trend of statistical significance (p = 0.052). In addition, Galectin-7 (*LGALS7*) has been identified as immunosuppressive in squamous cell carcinoma^35^ and therefore may also be of interest for future studies exploring potential molecules associated with immunosuppression in PDS. Lastly, upregulation of Complement C1q binding protein (C1QBP) was associated with decreased survival in breast cancer^36,37^; however, its exact biological role here remains to be determined.

Immunohistochemically, PDGFRB appeared to be uniformly expressed on PDS tumor cells (n = 41) but was not expressed in well-differentiated and undifferentiated cSCC tumors (n = 85)^1^. This indicates, that PDGFRB specifically discriminated between PDS and cSCC—implying a potential diagnostic value, which needs to be confirmed in additional studies. However, in the current study, only PDS tumors showed an activation of PI3K/AKT signaling, as determined by the analysis of receptor/ligand interactions of proteomic data, with a significant correlation between PDGFRB and PDGFD in PDS tumors, but not within MM or cSCC. This PDGFRB-PDGFD co-expression further strengthens the hypothesis that PDS may be of fibroblastic origin. However, given that we have analyzed macro dissected whole-tumor tissue sections, we cannot rule out that this signal comes from fibroblasts associated with the tumor microenvironment. But given that we have used several measures of tumor purity – both independently inferred from both DNA exome sequencing and RNA transcriptomic data – with similar results, we argue that our results do consider these potential issues of “signal noise” from surrounding tissue. It should be considered that Platelet-derived growth factor receptor beta (PDGFRB), is a tyrosine kinase receptor regulating cell proliferation, survival, and differentiation, whereas dysregulation of PDGFRB signaling has been implicated in the development and progression of a variety of cancers^38–40^. Several drugs have shown efficacy against PDGFRB, particularly small molecule tyrosine kinase inhibitors (TKIs) including imatinib, dasatinib, and nilotinib^41,42^. Potentially, these therapeutics might serve as an option for patients who have progressed under immunotherapy.

In conclusion, we have presented several lines of evidence to suggest a fibroblastic cell of origin in PDS using our proteomic analysis. We also highlight several proteins that were upregulated in PDS, compared to cSCC which may be studied for diagnostic value in the near future. Therapeutically, we also show that PDS can be divided into distinct subgroups, including immunologically “cold” tumors. Here, we highlight potential therapeutic options to convert "cold" tumors into secondary inflammatory “hot” tumors that may benefit from systemic PD-1 inhibition, which needs to be confirmed in additional studies.

## Materials and methods

### Patients

We analyzed 24 primary PDS tumors from 22 males and two females (mean age: 81 ± 6 years, range: 70–95 years). All 24 primary tumors were located on the head, specifically at the neurocranium, and were treatment naïve. Clinically, 18 of 24 patients (75%) had no tumor progression, whereas six patients (25%) showed a disease progression after a mean of 15 ± 7 months (range: 9–25 months), including two patients developing local relapses, one patient lymph node metastasis and three patients’ distant metastases (multiple subcutaneous metastases and lung metastases). In addition, we analyzed samples from 27 primary malignant melanomas (MM), 20 cutaneous squamous cell carcinomas (cSCC) and normal skin (n = 20).

### Inclusion criteria

For PDS cases, cases from the previous study^1^ with sufficient tissue were selected. To allow a comparison of different skin cancers and normal tissue with a similar power, we chose 27 MM cases – because we expected a higher variability within this entity, 20 cSCC, and 20 normal skin cases. MM, cSCC and normal skin were archival material of the Institute of Pathology and the Department of Dermatology (both University Hospital Cologne), diagnosed between 2017 and 2020. All diagnoses were reviewed by at least two pathologists following the WHO guidelines. Exclusion criteria were insufficient tissue or large area of necrosis within the tumor.

### Sample size and power calculations

For our retrospective study, we performed power calculations based on the primary outcome measures, particularly, these calculations were informed by review of the literature and empirical data from previous studies in similar patient and cancer type populations. A small to medium effect size (Cohen’s d) with an alpha level of 0.05 (5% significance level) and a power of 0.8 (80%) was used. For the power analysis we were using G*Power (3.1) software^43^. We expected a higher intertumoral heterogeneity in certain entities, such as malignant melanoma. Here, the power analysis indicated a required sample size of 27 subjects per group to adequately detect the specified effect size. For tumor types known to have less intertumoral/intersample heterogeneity, such as cutaneous squamous cell skin cancer and normal skin, a smaller effect size was anticipated. Consequently, the power analysis suggested that a sample size of 20 subjects per group would suffice to achieve the desired power level. This decision was further supported by prior studies in these specific tumor types, which typically demonstrated lower intra-group variance, thereby allowing for a reduction in the necessary sample size.

### Formalin-fixed paraffin-embedded -tissue lysis and digestion

Homogenization tubes were prepared by adding 200 µl of cooled phosphate buffered saline containing 1 × Halt Protease Inhibitor Cocktail (Thermo) to a mixture of 1.4 mm and 2.8 mm ceramic beads in 2 ml tubes (Bertin). Deparaffinization of the Formalin-Fixed Paraffin-Embedded (FFPE) samples was carried out using a standard procedure with the immersion of slides in xylene for three minutes, followed by fresh xylene for three minutes, and 100% ethanol twice, then 95% and 70% ethanol, each for one minute. The slides were then washed for 3 min in water. Subsequently, we performed a macro dissection – referring to a procedure of macroscopically dissecting Formalin-Fixed Paraffin-Embedded (FFPE) tumor carrying tissue whole tumor tissue specimens, each of 5 µm thickness and with an averaged tumor size of 10 mm × 10 mm. The tissue was added to the prepared tubes and homogenized with two 20 s cycles at 5800 rpm in a precooled Precellys ball mill (Bertin). After homogenization, the tubes were centrifuged for 5 min at 20.000×*g*, 4 °C. 190 μl supernatant was transferred to fresh 0.5 ml tubes. After addition of 50 µl 20% SDS (4% final) and 12 µl 100 mM dithiothreitol (DTT) (4.8 mM final) the samples were heated to 95 °C for 60 min and sonificated using a Bioruptor (Diagenode) with 10 × 30 s pulses and 30 s delay between pulses. The heating and sonification sequence was repeated one more time. To alkylate free sulfhydryl groups, 24.2 µl 400 mM chloroacetamide in water were added to a final concentration 35 mM and samples were incubated for 30 min at room temperature in the dark. A volume of 50 µl of protein lysates, corresponding to approximately 10 µg, was used for protein digestion according to the single-pot solid phase enhanced sample preparation protocol^44^. After the final peptide clean-up, the beads were resuspended in 9 µl of 5% DMSO. Ten minutes later, the magnetic beads were separated, and the clear supernatants were carefully transferred to new tubes. These supernatants were then acidified by adding 1 µl of 10% formic acid to ensure stabilization of the peptides for subsequent analyses.

### Mass spectrometry

Samples were analyzed on a Q Exactive Exploris 480 (Thermo Scientific) mass spectrometer equipped with a FAIMSpro differential ion mobility device that was coupled to an Ultimate 3000 RSLC nano lc system (Thermo Scientific). Approximately 2 µg of protein digests were loaded onto a trap column (µ-Prepcolumn, Thermo Scientific) in 0.1% trifluoroacetic acid in water and backflush eluted onto an in-house packed analytical column (30 cm–75 µm I.D., filled with 2.7 µm Poroshell EC120 C18, Agilent). Peptides were chromatographically separated at a constant flow rate of 300 nL/min and the following gradient: 2%B (0.1% formic acid in 80% acetonitrile) for 0.5 min, 2–6% B in 0.5 min, 2–32% B within 72.0 min, 32–55% solvent B within 7.0 min and 55–95% solvent B within 2.0 min, followed by a 6 min column wash with 95% solvent B and re-equilibration to 2% B for 5 min. The FAIMS pro was operated at -50 V compensation voltage and electrode temperatures of 99.5 °C for the inner and 85 °C for the outer electrode. Identical HPLC settings were used library generation and sample runs. Identical HPLC settings were used library generation and sample runs. Spectrum library generation by Gas phase fractionation^45^: Aliquots from each samples were pooled and the pool was used for spectrum library generation by narrow window DIA of six 100 m/z gas phase fractions (GPF) covering the range from 400 to 1000 m/z^45^. The Orbitrap was operated in DIA mode. MS1 scans of the respective 100 m/z gas phase fraction were acquired at 30 k resolution. Maximum injection time was set to 50 ms and the AGC target to 100%. MS2 scans of the corresponding 100 m/z region were acquired in 25 × 4 m/z staggered windows resulting in 50 nominal 2 m/z windows after demultiplexing. MS2 settings were 15 k resolution, 55 ms maximum injection time and an AGC target of 1000%. All scans were stored as centroids. Data independent acquisition of samples: MS1 scans were acquired from 390 to 1010 m/z at 15 k resolution. Maximum injection time was set to 25 ms and the AGC target to 100%. MS2 scans ranged from 300 to 1500 m/z and were acquired at 15 k resolution with a maximum injection time of 22 ms and an AGC target of 1000%. DIA scans covering the precursor range from 499.5 to 1000.5 m/z and were acquired in 60 × 11 m/z windows with 1 m/z overlap. All scans were stored as centroids.

### Data processing

The gas phase fractionation raw files were demultiplexed and transformed to mzML files using the msconvert module in Proteowizard. A human canonical Swissprot fasta file was transformed into a predicted spectral library using the “FASTA digestion for library free search “ and “Deep learning-based spectra, RT´s IM and IM´s prediction” options with the following settings in DIA-NN 1.8^46^: Trypsin specificity, up to 1 missed cleavage, precursor ion range 400–1000 m/z, fragment ion range 250–1500 m/z, charge states 2 + , 3 + and 4 + and default settings for the other parameters. The predicted library covering the entire human proteome (20,335 proteins, and 20,239 genes and 2,348,517 precursors) was searched with the six GPF runs and the “library generation” option in DIA-NN to generate an adjusted experiment specific spectral library (10,695 proteins, 10,661 genes and 79,370 precursors). As this spectral library contains only proteins that were detected in the gas phase fractionated all sample pool, it represents the entire experiment and was used for the final analysis of 91 sample runs. In addition to the settings used for library generation, grouping on protein names from fasta file, relaxed protein inference and retention time dependent normalization were used. Other options were left at default, including “matching between runs”. Although both MS1 and MS2 scans were performed, MS2 was used for inferring protein abundance. Proteins were quantified using MaxLFQ, as part of the DIA-NN R-package^46^. This method integrates peak intensities over multiple runs to estimate protein abundance. As a threshold for valid values proteins needed to have at least 70% valid values in at least one condition to be considered for further analysis. We normalized the data using a log2 transformation. To impute missing proteomics data, we used the imp4p R package [version 1.2]^47^, where we chose the entities as condition (PDS, MM, SNT, cSCC).The process involves using random forests to predict missing values based on patterns observed in the data, without a predefined threshold for determining when a value should be imputed. Proteomic data, also assigned to corresponding IDs of the previous study^1^, can be found in Table S1. The mass spectrometry proteomics raw data have been deposited to the ProteomeXchange Consortium via the PRIDE^48^ partner repository with the dataset identifier PXD046388.

### Data analysis

#### Estimation of tumor purity using RNA sequencing data

Processed, normalized mRNA sequencing data and metadata of the previously published PDS cohort were used and subsequently analyzed using quanTIseq^49^. Among estimated counts of cell types, quanTIseq provides an estimate of tumor purity for RNA sequencing data, which we used in addition to tumor purity calculated based on DNA exome sequencing data, as described previously^1^.

#### Correlation of tumor purity to protein abundance and overrepresentation analysis

Tumor purity was calculated using RNA- and DNA-sequencing data. Then, we correlated the protein abundance using a Spearman correlation approach by looping through the list of samples, correlating tumor purity as a measure and protein counts. We then averaged the individual correlations from both RNA- and DNA-derived tumor purity and ranked these correlations from highest to lowest (see Table S2). Subsequently, the top 50 highest-ranking correlations were used for overrepresentation analysis using Enrichr^11^ and dedicated gene sets (““GO Biological Process 2021”; “ARCHS4 tissue”) using the corresponding HUGO symbols.

#### Analysis of publicly available single cell data of skin normal tissue

We downloaded the merged primary data (GSE130973; healthy skin bulk tissue of five individuals) of the study ^12^ conducted by Solé-Boldo et al. For simplicity and reproducibility, we re-used the clusters and annotations that have been stored in the .rds file by the authors, and used Seurat (version 4.0.5)^50^ for subsequent analysis and visualization of the data.

#### Receptor and ligand interaction, pathway analysis

To uncover relevant receptor/ligand (R/L) interactions within the proteomic data of PDS (n = 24), MM (n = 27), and cSCC (n = 20), in addition to significant downstream signaling pathways, we used a previously published method, which was originally developed for single cell sequencing data and can now be applied to bulk-data^13,51,52^. Briefly, we calculated R/L interactions separately for each entity by converting the universal protein resource (UniProt) identifiers to human genome organization (HUGO) symbols, using the full dataset. Then, we combined the results and filtered using a threshold of 10^–3^ for the false discovery rate. A complete list of significant pairs of R/L interactions, together with associated Reactome pathways, can be found in Table S3.

### Statistical analysis

A p-value of less than 0.05 was considered statistically significant. A p-value between 0.1 and 0.05 was considered as a trend. All tests were two-sided. Statistical analysis was done with R (version 4.1.2) and Python (version 3.8.8).

### Ethics approval/consent to participate

The study protocol was conducted in accordance with the ethical guidelines of the 1975 Declaration of Helsinki. All investigations were performed after approval of the institution’s human research review committee of the University of Cologne, Germany (registration No. 15-307 and 20-1082). Informed written consent was obtained from each subject.

### Supplementary Information

Supplementary Figures.

Supplementary Table S1.

Supplementary Table S2.

Supplementary Table S3.

## Data Availability

Data supporting this study are included within the article and/or supporting materials.
