# Supplementary material for: Proteomic analysis of pleomorphic dermal sarcoma reveals a fibroblastic cell of origin and distinct immune evasion mechanisms
Source: Sci Rep. 2024 May 31;14:12516. doi: 10.1038/s41598-024-62927-x (PMC11143252; doi:10.1038/s41598-024-62927-x)
Supplement: Supplementary file 1 — Supplementary Figures. [file 41598_2024_62927_MOESM1_ESM.pdf]

Supplementary figures

Figure S1

a **b**

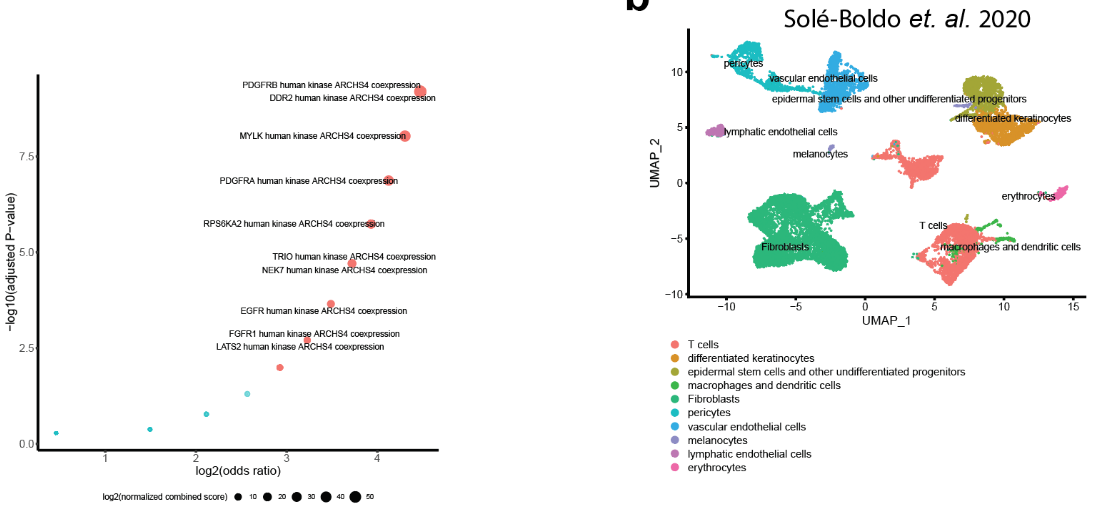

Figure S1 PDGFRB is associated with RNA/DNA purity and illustration of single cell sequencing data of a previously described dataset.

(A) Enrichr (Kuleshov et al., 2016) analysis using the “ARCHS4 kinase” database of the top 50 genes correlating to RNA/DNA purity. The color code represents significant findings (FDR < 0.05, red) (n=24). (**B**) UMAP of publicly available single cell sequencing data of normal skin tissue (Solé-Boldo et al., 2020). Individual cell clusters were used from the original paper and highlighted using a color code (n=5).

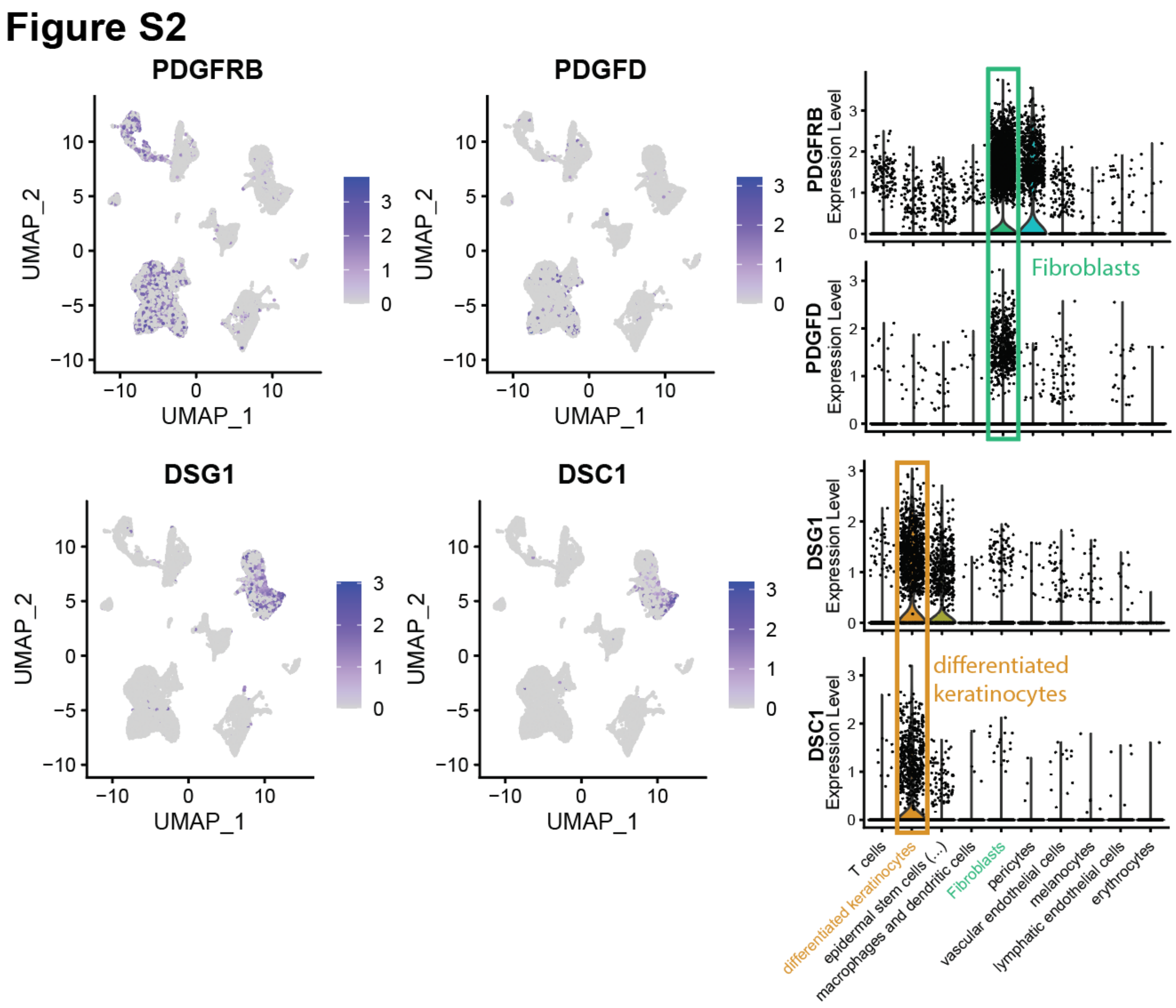

Figure S2. PDGFRB and PDGFRB expression is elevated on fibroblasts, while DSG1 and DSC1 is highly expressed on keratinocytes. UMAP of publicly available single cell sequencing data of normal skin tissue (Solé-Boldo et al., 2020) [corresponding cell types in Figure S1B] and violin plots showing expression of *PDGFRB*, *PDGFD*, *DSG1* and *DSC1* according to cell types.
